# RETRACTION: Effects of Electroacupuncture at Governor Vessel Acupoints on Neurotrophin‐3 in Rats with Experimental Spinal Cord Injury

**DOI:** 10.1155/np/9845020

**Published:** 2026-10-21

**Authors:** Neural Plasticity

RETRACTION: Y‐P. Mo, H‐J. Yao, W. Lv, et al., “Effects of Electroacupuncture at Governor Vessel Acupoints on Neurotrophin‐3 in Rats with Experimental Spinal Cord Injury,” *Neural Plasticity*, 2016, 2371875, https://doi.org/10.1155/2016/2371875.

The above article, published online on 11 August 2016 in Wiley Online Library (wileyonlinelibrary.com), has been retracted by agreement between the Journal Editor‐in‐Chief, Michel Baudry; and John Wiley & Sons Ltd. The retraction has been agreed due to concerns identified with the figures and the data, initially reported on PubPeer [1]. Specifically:

• The mean measurements and standard deviations in Table 1 are unexpectedly similar.
• In Figure 2, the 7d/C panel and the 3d/EA panels appear to be identical.
• In Figure 4, the GAPDH western blot panels appear identical to the GAPDH blots shown in Figure 8a of a later publication of some of the same authors [2], despite some of the blots corresponding to different timepoints.

The authors replied to an inquiry and provided some of the raw data. However, when their response was fully assessed by the Chief Editor, the results and conclusions were considered unreliable. The article is therefore retracted.

The authors do not agree to the wording of this retraction notice.

## References

[1] Bik E. , Effects of Electroacupuncture at Governor Vessel Acupoints on Neurotrophin-3 in Rats with Experimental Spinal Cord Injury, PubPeer, 2020, https://pubpeer.com/publications/F52FE24567D64E60838469FEDE868A. 10.1155/2016/2371875 PMC4997063 27597902

[2] Wang X. , Shi S. H. , and Yao H. J. , et al.Electroacupuncture at Dazhui (GV14) and Mingmen (GV4) Protects Against Spinal Cord Injury: The Role of the Wnt/β-catenin Signaling Pathway, Neural Regeneration Research. (2016) 11, no. 12, 2004–2011, 10.4103/1673-5374.197145. PMC5270441 28197199

